# Salidroside Promotes the Pathological α-Synuclein Clearance Through Ubiquitin-Proteasome System in SH-SY5Y Cells

**DOI:** 10.3389/fphar.2018.00377

**Published:** 2018-04-19

**Authors:** Tao Li, Yang Feng, Ruixin Yang, Leitao Wu, Ruru Li, Lu Huang, Qian Yang, Jianzong Chen

**Affiliations:** ^1^Research Center of Traditional Chinese Medicine, Xijing Hospital, The Fourth Military Medical University, Shaanxi, China; ^2^Department of Neurosurgery, Tangdu Hospital, The Fourth Military Medical University, Shaanxi, China

**Keywords:** Parkinson’s disease, salidroside, α-synuclein, UPS, 6-OHDA

## Abstract

Parkinson’s disease (PD) is characterized by the loss of dopaminergic (DA) neurons in the substantia nigra pars compacta (SNc) and the presence of Lewy bodies (LBs) in the surviving SNc neurons. LBs formation is caused by the accumulation of α-synuclein (α-syn) or phosphorylated α-syn at serine-129 (pSer129-α-syn), which is implicated in the pathological progression of PD. Salidroside (Sal), the main active ingredient of the root of *Rhodiola rosea L*., has been reported to have potent neuroprotective properties in our previous investigations. Here, we investigated the effects of Sal on 6-OHDA and overexpresssion of WT/A30P-α-syn-induced pathological α-syn increase and the mechanism behind it in SH-SY5Y cells. We found Sal displays neuroprotective effects against 6-hydroxydopamine (6-OHDA)-induced cytotoxicity. Sal decreased the pSer129-α-syn level mainly by maintaining the normal function of ubiquitin-proteasome system (UPS). Furthermore, Sal promoted the clearance of α-syn and protected the cell viability mainly through recovered the 20S proteasome activity in WT/A30P-α-syn-transfected cells. These data provide new mechanistic insights into the neuroprotective effects of Sal and Sal may be a promising therapy to slow neurodegeneration in PD.

**Highlights:** Sal protects cells and decreases the pSer129-α-syn protein level in 6-OHDA-induced impairmental and dysfunctional SH-SY5Y cells.

Sal promotes the clearance of α-syn and protects the cell viability mainly through recovering the 20S proteasome activity in WT/A30P-α-syn plasmids transfected cells.

Maintaining the normal function of the UPS may be one of the important mechanisms of Sal in neuroprotective effects.

## Introduction

Parkinson’s disease (PD) is the second most common neurodegenerative disorder. The pathological hallmarks of PD are the loss of dopaminergic (DA) neurons in the substantia nigra pars compacta (SNc) and the presence of Lewy bodies (LBs) in the surviving SNc neurons. LBs are mainly composed of α-synuclein (α-syn) (Spillantini et al., 1997; Goedert et al., 2013). In normal neurons, α-syn is thought to be involved in regulating the dynamics, trafficking of synaptic vesicles and neurotransmitter release (Clayton and George, 1999; Zhang L. et al., 2008). Studies suggested that approximately 90% of the α-syn deposited in LBs is phosphorylated at Ser-129 (pSer129-α-syn) (Fujiwara et al., 2002; Anderson et al., 2006). Postmortem studies of human α-synucleinopathies and animal PD models have shown that the level of pSer129-α-syn is linked to the disease progression (Braithwaite et al., 2012; Sato et al., 2013). Missense point mutations in the *SNCA* gene, such as A53T or A30P, cause autosomal-dominant early-onset PD (Polymeropoulos et al., 1997; Kruger et al., 1998). Investigations of A53T/A30P transgenic mouse models have shown that the accumulation of A53T/A30P-α-syn resulted in the loss of SNc neurons, dysfunction of DA neurons, and behavioral impairments (Freichel et al., 2007; Paumier et al., 2013). Therefore, the accumulation of pathological α-syn is likely involved in the neuronal degeneration observed in PD and it may be a potential target for therapeutic intervention.

The ubiquitin-proteasome system (UPS) and autophagy-lysosome pathway (ALP) are the two major degradation pathways in eukaryotic cells. They are tightly connected, modulating proteome homeostasis and quality in response to physiological cues and environmental stresses (Cohen-Kaplan et al., 2016). Moreover, α-syn inhibits proteasome activity, while other members of the synuclein family regulate proteasomal function (Snyder et al., 2005). Dysfunction of the UPS induced by proteasome inhibitors, pesticides, or genetic causes leads to the degeneration and death of DA neurons and the accumulation of α-syn-positive inclusion bodies (McNaught and Olanow, 2006; Wang et al., 2006; Shimshek et al., 2012). This suggests that maintaining normal function of the UPS may be a promising strategy to remove the pathological α-syn and protect DA neurons.

**FIGURE 1:**
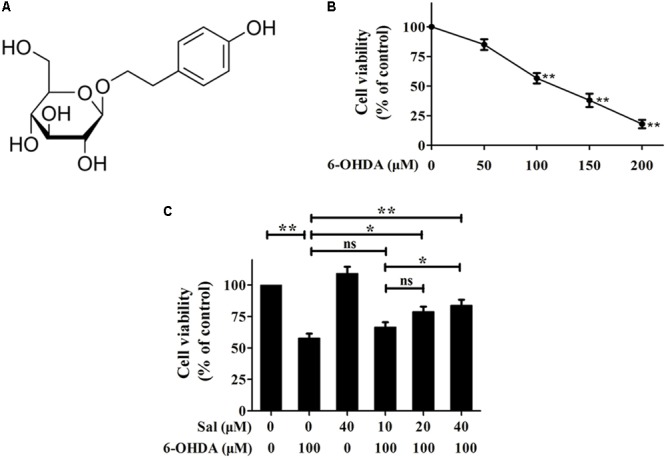
Protective effects of Sal against 6-OHDA induced cytotoxicity in SH-SY5Y cells. **(A)** Sal is glucoside of tyrosol (p-hydroxyphenethyl-β-D-glucoside). **(B)** 6-OHDA treatment reduces cell viability of SH-SY5Y cells. SH-SY5Y cells were incubated with various concentrations (50, 100, 150, and 200 μM) of 6-OHDA for 24 h then the cell viability was assayed using MTT. **(C)** Sal attenuates 6-OHDA-induced reduction in cellular viability. Cells were pretreated with various concentrations (10, 20, and 40 μM) of Sal for 24 h after which 6-OHDA (100 μM) was added for an additional 24 h. The cell viability was measured using MTT. Data are shown as the mean ± SEM. Statistical significance was analyzed by one-way analysis of variance (ANOVA). A two-tailed Student’s *t*-test was performed for comparisons between two groups. Each experiment was repeated three times independently. ^∗^*P* < 0.05; ^∗∗^*P* < 0.01, ns: no significance.

Salidroside [p-hydroxyphenethyl-β-D-glucoside (Sal)] is the primary active ingredient in the roots of *Rhodiola rosea L*., a popular plant used in traditional medicine in Asian and Eastern European countries (**Figure 1A**). It has been reported to have a variety of pharmacological effects, including anti-inflammatory, anti-oxidative, anti-fatigue, anti-aging, and neuroprotective effects (Zhang et al., 2013; Chen et al., 2015; Ma et al., 2015; Jin et al., 2016).

Our previous studies revealed that Sal protected against 1-methyl-4-phenylpyridinium (MPP^+^)/1-methyl-4-phenyl-1,2,3,6-tetrahydropyridine (MPTP)-induced PD model through ROS-NO-related mitochondria pathway *in vitro* and *in vivo* and found Sal promoted the α-syn clearance, however, the behind mechanism is unclear (Wang et al., 2015). Here, we used 6-hydroxydopamine (6-OHDA) and overexpression of WT/A30P-α-syn to artificially increase the intracellular levels of α-syn, then explored the effects of Sal on pathological α-syn. We found Sal protected cells against 6-OHDA-induced cytotoxicity and reduced pSer129-α-syn level. Sal achieved this mainly by protecting the function of the UPS. Finally, we found that Sal promoted the clearance of α-syn and protected SH-SY5Y cells that overexpressed WT/A30P-α-syn by recovering the activity of 20S proteasome. These results indicate that Sal may be a potential neuroprotective agent that promotes the pathological α-syn clearance and slows neurodegeneration in PD.

## Materials and Methods

### Materials

Sal (> 99.4% purity) was purchased from the National Institute for the Control of Pharmaceutical and Biological Products (Xi’an, China). 6-OHDA, ascorbic acid, momotetrazolium (MTT), and dimethyl sulfoxide were purchased from Sigma–Aldrich (St. Louis, MO, United States). Dulbecco’s modified Eagle’s medium and heat-inactivated fetal calf serum were purchased from Corning Cellgro (United States). A proteasome activity assay kit (APT280) was purchased from Merk Millipore (United States). MG132 was purchased from MedChem Express (United States). Hydroxychloroquine sulfate (HCQ) was purchased from Selleckchem (United States). PGL3-CMV-LUC-MCS (PGL3) was purchased from Genomeditech (#2438, China). Luciferase Reporter Gene Assay kit was bought from Roche (#11814036001, Switzerland). The following antibodies were purchased from Cell Signaling Technology (United States): anti-PARKIN (1:1000, #4211), anti-LC3-II (1:1000, #4108), anti-UCH-L1 (1:1000, D3T2E), and anti-α-syn (1:1000, #2628). The following antibodies were obtained from Sigma–Aldrich (United States): anti-p62 (1:5000, ab109012) and anti-pSer129-α-syn (1:1000, EP1536Y). The following antibody was obtained from Abcam (United States): anti-ubiquitin (1:1000, U0508). The following antibody was bought from Merk Millipore (United States): anti-20S proteasome core subunit (1:1000, ST1053). The following antibodies were obtained from Sangon Biotech (China): anti-β-ACTIN (1:3000, D110024), HRP-conjugated goat anti-rabbit IgG (1:5000, D110058), and HRP-conjugated goat anti-mouse IgG (1:5000, D110087). Lipofectamine 2000 Transfection Reagent and Alexa Fluor^®^ 488 Goat Anti-rabbit IgG secondary antibody were purchased from Invitrogen (United States).

### Cell Culture and Treatment

SH-SY5Y cells were cultured in Dulbecco’s modified Eagle medium (Corning Cellgro, United States) supplemented with 10% heat-inactivated fetal calf serum (Corning Cellgro, United States), 1% penicillin (100 IU/ml), and 1% streptomycin (100 mg/ml) under 5% CO_2_ at 37°C. Cells were seeded onto poly-L-lysine-coated plates and passaged when cells reached 60–70% confluence. Cells were pretreated for 24 h with or without different concentrations of Sal (10, 20, or 40 μM) before the addition of 6-OHDA (100 μM) for an additional 24 h. Cells were divided into a control group, 6-OHDA-treated group, Sal-treated group, and a group pretreated with different concentrations of Sal (10, 20, or 40 μM) followed by 6-OHDA treatment.

### Measurement of Cell Viability

Cell viability was determined using the MTT assay (Sigma–Aldrich, United States) according to the manufacturer’s instructions. Briefly, SH-SY5Y cells were seeded into a 96-well plate at a concentration of 1 × 10^4^/well. After an overnight incubation, plates were first incubated with Sal (National Institute for the Control of Pharmaceutical and Biological Products, China) for 24 h, then the Sal solution was removed and washed three times with PBS. Subsequently, cells were treated with 6-OHDA (Sigma–Aldrich, United States) or PBS for another 24 h. A total of 20 μL of MTT (5 mg/mL) was added to each well and incubated at 37°C for 4 h. The medium was then removed, and 150 μL of dimethyl sulfoxide was added to each well. For cell viability assay in transfected cells, SH-SY5Y cells were seeded into a 48-well plate. After cells transfection and Sal or Sal+MG132 treatment, 40 μL of MTT (5 mg/mL) was added to each well and incubated at 37°C for 4 h. The medium was then removed, and 300 μL of dimethyl sulfoxide was added to each well. After shaking for 10 min, the absorbance at 570 nm was measured in a microplate reader (Bio-Rad, Hercules, CA, United States). Cell viability was expressed as a percentage of that of the control group.

### Western Blotting

Treated cells were collected and lysed in lysis buffer (50 mM Tris-HCl, 150 mM NaCl, 0.02% NaN_2_, 100 μg/ml phenylmethanesulfonyl fluoride, 1 μg/ml aprotinin, and 1% Triton X-100) in the presence of protease inhibitors and phosphatase inhibitors on ice. The lysate was centrifuged at 13,000 rpm for 10 min, and the supernatant was used for analysis. Protein concentrations were determined using the Bradford assay kit (Bio-Rad Laboratories, United States). The lysed samples were separated by SDS–PAGE and then transferred to polyvinylidene fluoride membranes. The membranes were blocked with 5% skim milk for 2 h at room temperature and then incubated with specific primary antibodies with gentle shaking at 4°C overnight. After washing, the membranes were incubated with HRP-conjugated secondary antibodies for 2 h. Protein bands were visualized using enhanced chemiluminescence. Protein quantifications were obtained using Image J software (National Institutes of Health, Bethesda, MD, United States).

### Immunocytochemistry

For immunocytochemistry, SH-SY5Y cells were grown on poly-L-lysine-coated slides and fixed with 4% paraformaldehyde (adjusted to pH 7.4) for 15 min. Cells were permeabilized with 0.1% Triton X-100 for 30 min and blocked in 2% bovine serum albumin in phosphate-buffered saline (PBS) for 1 h at room temperature. After washing with PBS, the cells were incubated at 4°C overnight with an antibody against pSer129-α-syn. The next day, the cells were washed with PBS and labeled with an anti-rabbit secondary antibody conjugated to Alexa-Fluor 488 (1:200 dilution) for 2 h at room temperature. Nuclei were labeled with DAPI for 10 min. Final images were acquired using a laser-scanning confocal microscope (A1plus; Nikon, Japan). The pSer129-α-syn fluorescent images were captured with a 40 × object lens in emission wavelength of 525 nm and excitation wavelength of 488 nm (laser power 3.4, PMT HV 100, offset-7). The nucleus images stained by DAPI were acquired with a 40 × object lens in emission wavelength of 450 nm and excitation wavelength of 405 nm (laser power 3.4, PMT HV 99, offset-5). The mean fluorescence intensities were further analyzed by Image-Pro Plus 6.0 and expressed as a ratio to control.

### Proteasome Activity Assay

Proteasome activity was measured using the 20S Proteasome Activity Assay kit (APT280 Millipore, United States) according to the manufacturer’s instructions. Briefly, SH-SY5Y cells were lysed in lysis buffer [50 mM HEPES (pH 7.5), 5 mM EDTA, 150 mM NaCl, and 1% Triton X-100]. The assay mixture was prepared in a 96-well fluorometer plate, and samples were incubated for 1–2 h at 37°C. Fluorescence was measured at an excitation of 380 nm and an emission of 460 nm in a fluorometer. The 20S proteasome activity was expressed as a percentage of that of the control group.

### α-Syn Plasmid Transfection

Human WT/A30P-α-syn plasmids were gifts from Professor Yang Qian (Department of Neurosurgery, Tangdu Hospital, Fourth Military Medical University, Xi’an, China). Cells were transfected with WT/A30P-α-syn plasmid or the control pcDNA3 plasmid for 24 or 48 h, respectively, using Lipofectamine 2000 according to the manufacturer’s instructions.

### Luciferase Reporter Gene Assay

For CMV promoter transcription activity analyses in pcDNA3 vector, SH-SY5Y cells were transfected with PGL3-CMV-LUC-MCS (PGL3, #2438, Genomeditech, China), which containing the CMV promoter and luciferase reporter gene. After 24 h, cells were treated with DMSO or MG132 (0.25 μM) for another 24 h and lysed. An aliquot of the cell lysates were analyzed for luciferase activity using the Luciferase Reporter Gene Assay kit (#11814036001, Roche, Switzerland). Cells were harvested and treated with 400 μL of lysis reagent for 20 min, centrifuged 12000 ×*g* for 5 min. A total of 100 μL of supernatant from each sample was placed into a new microplate, and 100 μL of luciferase assay reagent was added to each well. The plate was immediately analyzed using the microplate reader (Bio-Rad, United States) at an absorbance of 570 nm.

### Statistical Analysis

All statistics were analyzed in SPSS software 14.0 (IBM Inc., Armonk, NY, United States). Data are presented as the means ± SEM and the differences among the control and various treatment groups were compared using one-way analysis of variance (ANOVA). Two-tailed Student’s *t*-test was used as a *post hoc* test to assess differences between two groups. *P* < 0.05 was considered statistically significant.

## Results

### Sal Pretreatment Attenuated 6-OHDA-Induced Cytotoxicity in SH-SY5Y Cells

We first performed a dose–response experiment to determine the optimal concentration of 6-OHDA to use on cells. Cell viability was assayed using the MTT assay after cells were exposed to various concentrations of 6-OHDA (50, 100, 150, and 200 μM) for 24 h. The results showed that cells treated with 100 μM of 6-OHDA for 24 h displayed a 56.7 ± 4.4% drop in viability compared to the control group. Therefore, a concentration of 100 μM was used for 6-OHDA treatments in subsequent experiments (**Figure 1B**).

We next determined whether pretreatment with Sal had protective effects against 6-OHDA-induced cell death. After cells were treated with 10, 20, or 40 μM of Sal for 24 h, 100 μM of 6-OHDA was added for another 24 h of culture. Sal suppressed 6-OHDA-induced cytotoxicity in a dose-dependent manner. Cell viabilities recovered to 66.3 ± 4.1% (10 μM Sal), 78.7 ± 4.2% (20 μM Sal), and 83.7 ± 4.7% (40 μM Sal) that of the control (**Figure 1C**).

### Sal Pretreatment Decreased pSer129-α-Syn Level Following 6-OHDA Treatment in SH-SY5Y Cells

**FIGURE 2:**
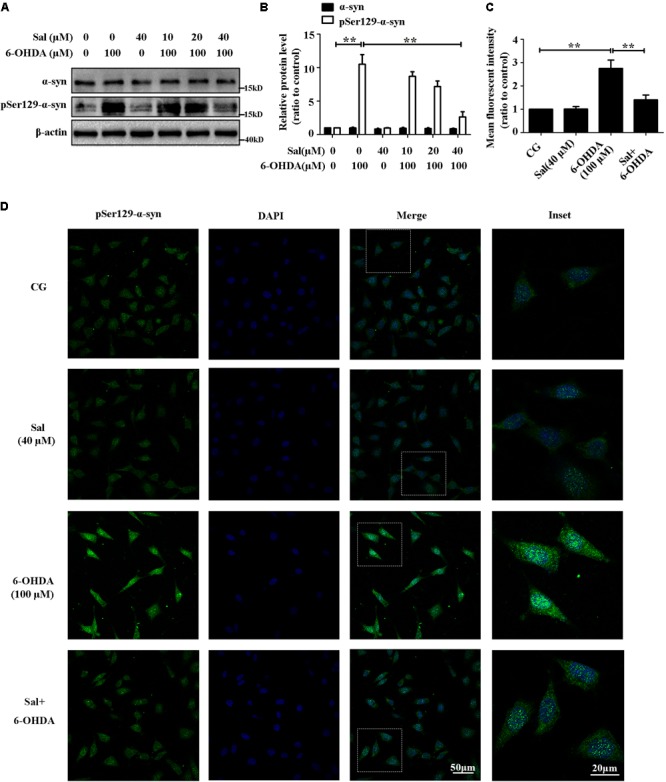
Sal pretreatment decreased pSer129-α-syn protein level against 6-OHDA-induced increase in SH-SY5Y cells. **(A)** Western blot analysis of α-syn and pSer129-α-syn protein levels in SH-SY5Y cells. Cells were preincubated with Sal (10, 20, and 40 μM) for 24 h after which 6-OHDA (100 μM) was added for another 24 h. **(B)** Graph shows the quantification of α-syn and pSer129-α-syn protein levels. **(C** and **D)** Immunofluorescence analysis of pSer129-α-syn in SH-SY5Y cells. Cells were preincubated with Sal (40 μM) for 24 h after which 6-OHDA (100 μM) was added for an additional 24 h. **(C)** Quantitative analysis of the pSer129-α-syn immunofluorescence intensity. **(D)** The pSer129-α-syn protein level were analyzed using immunofluorescence (green: pSer129-α-syn; blue: DAPI; the insets represent the boxed areas), bar: 50 μm. Data are shown as the mean ± SEM. Statistical significance was analyzed by one-way analysis of variance (ANOVA). A two-tailed Student’s *t*-test was performed for comparisons between two groups. Each experiment was repeated three times independently. ^∗^*P* < 0.05; ^∗∗^*P* < 0.01, ns: no significance.

The LBs are mainly composed of pSer129-α-syn in SNc neurons, and these are the major pathological feature of PD. In this study, we found that 6-OHDA (100 μM) highly increased the protein level of pSer129-α-syn without affecting the total protein level of α-syn in SH-SY5Y cells. Cells were pretreated with Sal (10, 20, or 40 μM) for 24 h, and then exposed to 100 μM of 6-OHDA for another 24 h. Western blotting (**Figures 2A,B**) and immunocytochemistry assays (**Figures 2C,D**) showed that pretreatment with 40 μM of Sal decreased the pSer129-α-syn protein level induced by 6-OHDA.

### Sal Protected the UPS from 6-OHDA-Induced Dysfunction in SH-SY5Y Cells

**FIGURE 3:**
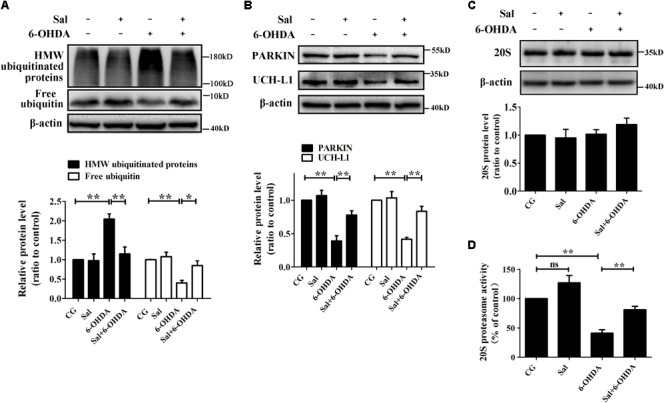
Sal protected the UPS from 6-OHDA-induced dysfunction in SH-SY5Y cells. Cells were preincubated with Sal (40 μM) for 24 h, then exposed to 6-OHDA (100 μM) for another 24 h. Immunoblot levels of highly molecular weight (HMW)-ubiquitinated proteins, free ubiquitin **(A)**, PARKIN, Ubiquitin C-terminalhydrolase-L1 UCH-L1 **(B)**, and 20S Proteasome core subunits **(C)**; **(D)** Proteasome activity assay analysis of 20S proteasome activity. Data are shown as the mean ± SEM. Statistical significance was analyzed by one-way analysis of variance (ANOVA). A two-tailed Student’s *t*-test was performed for comparisons between two groups. Each experiment was repeated three times independently. ^∗^*P* < 0.05; ^∗∗^*P* < 0.01, ns: no significance.

To clarify how Sal modulates pSer129-α-syn protein level, we examined its effects on marker proteins of UPS and proteasome activity in the 6-OHDA-induced PD model. We found that 6-OHDA (100 μM) treatment decreased free ubiquitin, PARKIN, and ubiquitin C-terminal hydrolase L1 (UCH-L1) protein levels to 40.4 ± 6.3%, 38.9 ± 7.9%, and 41.6 ± 2.7% compared to the control group, respectively. The 6-OHDA increased the amount of high molecular weight ubiquitinated proteins (a sign of proteasomal damage) to 204.6 ± 13.2% compared to the control group (**Figures 3A,B**). Moreover, 6-OHDA treatment reduced 20S proteasome activity to 41.0 ± 6.2% of the control (**Figure 3D**) without affecting the overall protein levels of the 20S proteasome (**Figure 3C**). However, cells pretreated with Sal for 24 h and then exposed to 6-OHDA for 24 h, showed a 114.8 ± 18.1% decrease in ubiquitinated high molecular weight proteins compared to the 6-OHDA-treated group and a increase in the protein levels of free ubiquitin, PARKIN, and UCH-L1. Sal pretreatment also rescued the activity of the 20S proteasome, significantly increasing it to 81.0 ± 6.1% compared to the 41.0 ± 6.2% activity level of the 6-OHDA-treated group (**Figure 3D**).

### Sal Decreased pSer129-α-Syn Protein Level Mainly Through UPS in SH-SY5Y Cells

**FIGURE 4:**
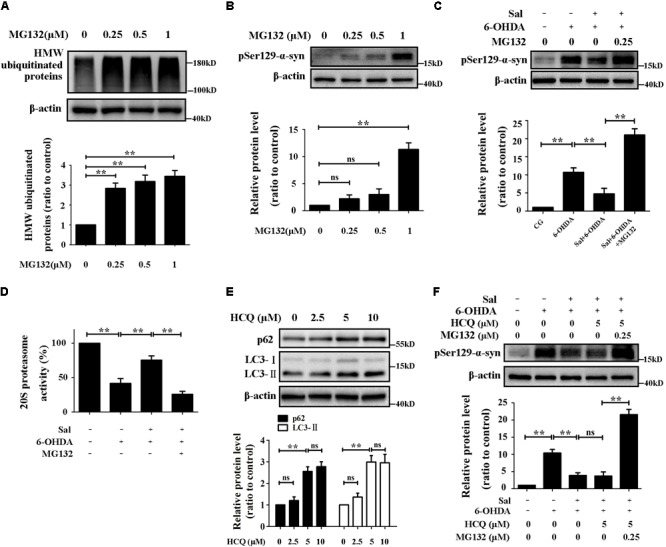
Sal decreased pSer129-α-syn protein level mainly through UPS in SH-SY5Y cells. **(A,B)** Immunoblot levels of HMW-ubiquitinated proteins and pSer129-α-syn after inhibited the UPS by various concentrations of MG132 (0.25, 0.5, and 1 μM) in SH-SY5Y cells. **(C)** Western blot analysis of pSer129-α-syn and PLK2 protein levels in SH-SY5Y cells. Cells were preincubated with Sal (40 μM) for 24 h, then exposed to 6-OHDA another 24 h in the presence or absence of MG132. **(D)** Proteasome activity assay analysis of 20S proteasome activity. Cells were preincubated with Sal (40 μM) for 24 h, then exposed to 6-OHDA another 24 h in the presence or absence of MG132. **(E)** The p62 (a selective substrate of macroautophagy) and LC3-II (autophagosome marker) protein levels were analyzed by Western blotting. Cells were incubated with HCQ (autophagy inhibitor) for 24 h. **(F)** Immunoblot level of pSer129-α-syn in SH-SY5Y cells. Cells were preincubated with Sal (40 μM) for 24 h, then exposed to 6-OHDA for another 24 h in the presence or absence of HCQ, HCQ, and MG132 in SH-SY5Y cells. Data are shown as the mean ± SEM. Statistical significance was analyzed by one-way analysis of variance (ANOVA). A two-tailed Student’s *t*-test was performed for comparisons between two groups. Each experiment was repeated three times independently. ^∗^*P* < 0.05; ^∗∗^*P* < 0.01, ns: no significance.

MG132, an inhibitor of the chymotrypsin-like proteasome activity inhibitor, was used to downregulate UPS activity. We treated cells with different concentrations of MG132 (0.25, 0.5, or 1 μM) for 24 h, and found that 0.25 μM of MG132 was suitable for our experiments (**Figures 4A,B**), which concentration of MG132 inhibited the proteasome activity, increased high molecular weight ubiquitinated-proteins but did not affect the pSer129-α-syn level. We pretreated cells with Sal for 24 h, then exposed them to 6-OHDA or 6-OHDA and MG132 for another 24 h. The results in **Figure 4C** showed that pSer129-α-syn protein level in cells pretreated with Sal was significantly lower compared to the 6-OHDA-treated group. Furthermore, 20S proteasome activity recovered to 75.3 ± 6.4% (**Figure 4D**) in the Sal-pretreated group compared to the activity of the 6-OHDA-treated group (41.7 ± 6.9%). After proteasome activity was inhibited by MG132, the effect of Sal on pSer129-α-syn protein level was reversed following disruption of the UPS.

The HCQ, a lysosomal acidification inhibitor and autophagic flux blocker, was used to inhibit the autophagy (Cook et al., 2014). After cells were treated with different concentrations of HCQ (2.5, 5, or 10 μM) for 24 h, the expression levels of LC3-II and p62, markers of autophagy activity, were assessed by Western blotting. The results showed that 5 μM of HCQ was suitable for use in subsequent experiments (**Figure 4E**). Cells were pretreated with Sal for 24 h first, then they were exposed to 6-OHDA, or 6-OHDA with HCQ, or 6-OHDA with HCQ and MG132, respectively, for another 24 h. Autophagy inhibition had no significant effect on pSer129-α-syn protein levels following Sal treatment (**Figure 4F**). However, after inhibiting both autophagy and the UPS, the pSer129-α-syn protein level significantly increased following Sal treatment.

### Sal Promoted α-Syn Clearance and Recovered 20S Proteasome Activity in WT/A30P-α-Syn-Transfected SH-SY5Y Cells

**FIGURE 5:**
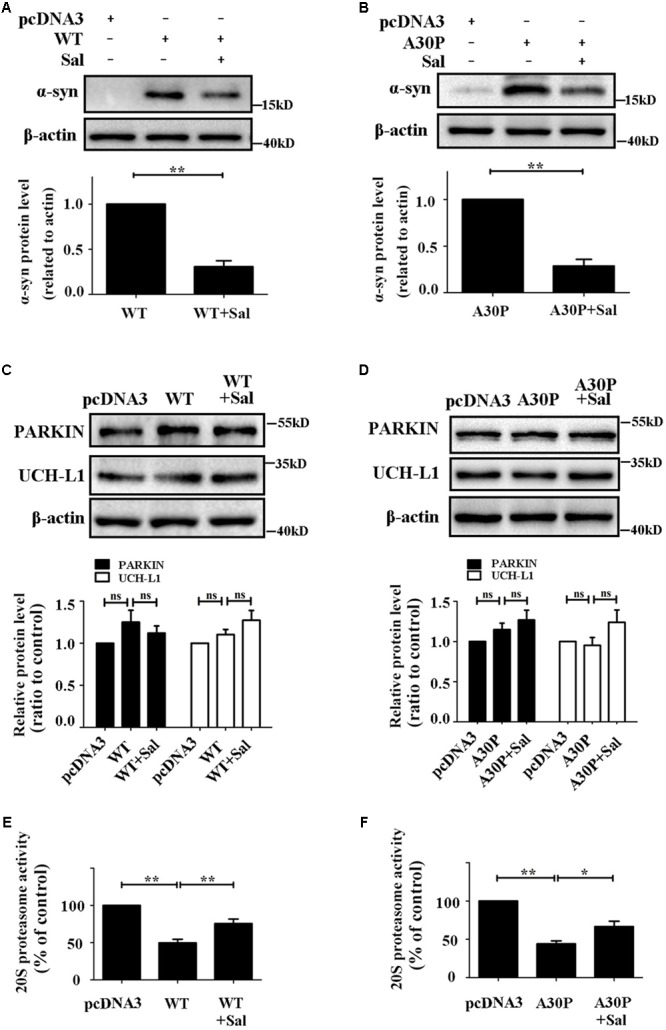
Sal promoted α-syn clearance and recovered the 20S proteasome activity in WT/A30P-α-syn-transfected SH-SY5Y cells. SH-SY5Y cells were transfected with WT/A30P-α-syn plasmids for 24 h after which Sal (40 μM) was added for an additional 24 h. **(A,B)** Western blot analysis of α-syn protein levels. **(C,D)** Immunoblot levels of PARKIN and UCH-L1. **(E,F)** Proteasome activity assay analysis of 20S proteasome activity. Data are shown as the mean ± SEM. Statistical significance was analyzed by one-way analysis of variance (ANOVA). A two-tailed Student’s *t*-test was performed for comparisons between two groups. Each experiment was repeated three times independently. ^∗^*P* < 0.05; ^∗∗^*P* < 0.01, ns: no significance.

We investigated the α-syn protein levels after cells were transfected with WT/A30P-α-syn plasmids and cultured for 24 and 48 h and found that α-syn protein was stably expressed between 24 and 48 h (Supplementary Figure 1). Furthermore, endogenous α-syn was nearly undetectable compared to the WT/A30P-α-syn protein produced by the overexpression construct. Therefore, in the subsequent experiments, the α-syn observed in our assays was considered to be the exogenously overexpressed WT/A30P-α-syn. We next transfected SH-SY5Y cells with WT/A30P-α-syn plasmids for 24 h, and then treated the cells with Sal (40 μM) for another 24 h. This revealed that Sal treatment reduced overall WT/A30P-α-syn protein levels to 30.7 ± 6.7 and 28.7 ± 7.1%, respectively (**Figures 5A,B**). To determine whether WT/A30P-α-syn clearance following Sal treatment was mediated by the UPS, we tested the protein level of PARKIN and UCH-L1, and 20S proteasome activity. Sal treatment had no significant influence on PARKIN and UCH-L1 protein levels compared to the WT/A30P-α-syn-transfected group (**Figures 5C,D**). Furthermore, after WT/A30P-α-syn transfection, 20S proteasome activity decreased to 49.7 ± 4.9 and 44.0 ± 4.1%, respectively (**Figures 5E,F**). Compared to the WT/A30P-α-syn-transfected group, treatment with Sal for 24 h increased 20S proteasome activity to 75.7 ± 6.1 and 66.7 ± 7.0%.

### Sal Protected Cells and Promoted α-Syn Clearance by Enhancing 20S Proteasome Activity in WT/A30P-α-Syn-Transfected Cells

**FIGURE 6:**
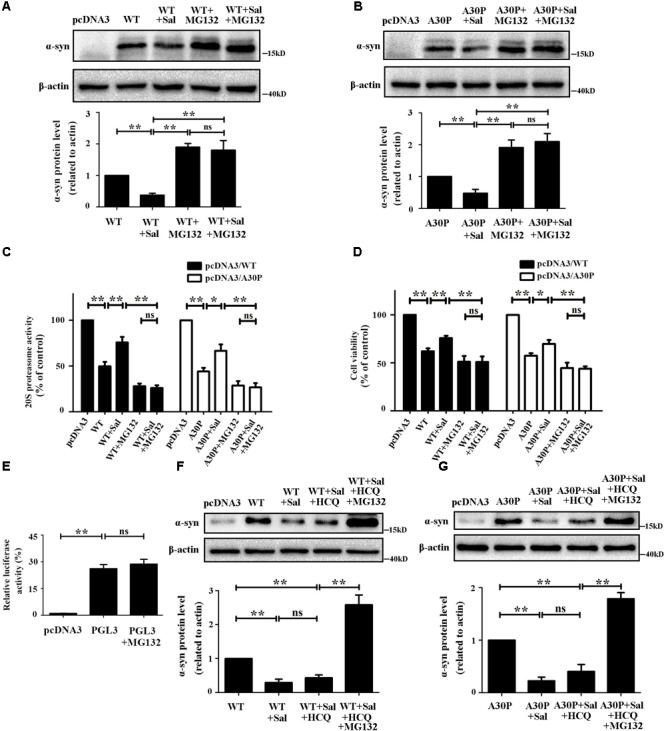
Sal protected cells and promoted α-syn clearance by enhancing 20S proteasome activity in WT/A30P-α-syn-transfected cells. SH-SY5Y cells were transfected with WT/A30P-α-syn for 24 h after which Sal (40 μM) was added for an additional 24 h in the presence or absence of MG132. **(A,B)** Immunoblot levels of WT/A30P-α-syn in SH-SY5Y cells. **(C)** Proteasome activity assay analysis of 20S proteasome activity. **(D)** MTT assay analysis of cell viability in SH-SY5Y cells. **(E)** Luciferase reporter gene assay. SH-SY5Y cells were transfected with pcDNA3 vector and PGL3 for 24 h, then the cells treated with MG132 (0.25 μM) or DMSO for another 24 h. **(F,G)** Western blot analysis of WT/A30P-α-syn protein levels in SH-SY5Y cells. Cells were transfected with WT/A30P-α-syn for 24 h after which Sal (40 μM) was added for an additional 24 h in the presence or absence of HCQ, HCQ, and MG132. Statistical significance was analyzed by one-way analysis of variance (ANOVA). A two-tailed Student’s *t*-test was performed for comparisons between two groups. Data are shown as the mean ± SEM. Each experiment was repeated three times independently. ^∗^*P* < 0.05; ^∗∗^*P* < 0.01, ns: no significance.

To confirm whether WT/A30P-α-syn clearance following Sal treatment was mediated by the UPS, we transfected cells with WT/A30P-α-syn plasmid for 24 h. Cells were then treated with the vehicle, Sal (40 μM), MG132 (0.25 μM), or both Sal (40 μM) and MG132 (0.25 μM) for another 24 h. MG132 treatment alone increased the WT/A30P-α-syn to 190.0 ± 11.6 and 191.3 ± 23.8%, respectively. The cells treated with Sal and MG132 displayed a significant increase in WT/A30P-α-syn protein levels of 180.0 ± 30.5 and 210.0 ± 25.2%, respectively, compared to the controls (**Figures 6A,B**). Besides, compared with obvious decrease of 20S proteasome activity in WT/A30P-α-syn transfected-group, treatment with Sal for 24 h recovered 20S proteasome activity to 71.3 ± 3.8 and 64.0 ± 6.7%. However, after cells were treated with MG132 or both Sal and MG132, 20S proteasome activity showed a significant decrease (**Figure 6C**). Moreover, Sal treatment for 24 h increased cell viability to 75.9 ± 2.3 and 69.7 ± 4.1%. In contrast, MG132 alone or both Sal and MG132 treatment displayed a significant decrease in the cell viability (**Figure 6D**).

To clarify the MG132 treatment increased the expression of α-syn by inhibiting the proteasome but not by activating the CMV promoter present in pcDNA3 vector. We performed the Luciferase reporter gene assay using vector-PGL3-CMV-LUC-MCS (PGL3). The cells were transfected with pcDNA3 vector and PGL3 for 24 h, then the cells treated with MG132 (0.25 μM) or DMSO for another 24 h. The result showed that 0.25 μM of MG132 has no significant effect on CMV promoter (**Figure 6E**). We then assessed the effect of Sal on α-syn protein levels in autophagy inhibition after WT/A30P-α-syn transfection. The results showed that autophagy inhibition had no significant influence on the α-syn clearance mediated by Sal (**Figures 6F,G**). Cells treated with Sal for 24 h and then exposed to inhibitors of autophagy and proteasome activity showed WT/A30P-α-syn protein level increases of 258.7 ± 28.9 and 179.0 ± 11.6%, respectively.

## Discussion

In the present study, we investigated the potential effects of Sal on the pathological increase in α-syn induced by 6-OHDA and WT/A30P-α-syn transfection in SH-SY5Y cells and the mechanisms underlying this biology. Interestingly, we found Sal alleviated 6-OHDA-induced cytotoxicity and decreased the pSer129-α-syn protein level via the UPS. Sal maintained the normal function of the UPS by recovering the protein levels of UCH-L1, PARKIN, free ubiquitin, and 20S proteasome activity. Furthermore, we found Sal protected cells and promoted α-syn clearance in WT/A30P-α-syn-transfected cells mainly through recovering 20S proteasome activity.

The LBs, which accumulate in SNc neurons, are the major pathological feature of PD. They are mainly composed of α-syn. Accumulating evidence suggests that several posttranslational modifications of α-syn, such as phosphorylation, ubiquitination, nitration, oxidation, and dopamine-modified formation, are toxic to SNc neurons in PD (Paxinou et al., 2001; McFarlane et al., 2005; Smith et al., 2005; Martinez-Vicente et al., 2008). Immunohistochemical and biochemical studies have shown that approximately 90% of the α-syn deposited in LBs is pSer129-α-syn. In normal brains, only 4% of the total α-syn is phosphorylated, which suggests that pSer129-α-syn might be involved in the formation of LBs and the pathogenesis of PD (Fujiwara et al., 2002). In our study, 6-OHDA elicited dose-dependent cytotoxicity in SH-SY5Y cells and Sal could inhibit the pSer129-α-syn accumulation induced by 6-OHDA. The 6-OHDA causes oxidative stress, mitochondrial dysfunction and finally results in the damage or apoptosis of DA neurons (Hanrott et al., 2006). PARKIN, which translocation from cytosol to mitochondria is an initial step in the mitophagy process, regulates mitochondrial quality control and protein ubiquitination. Our study showed that Sal increased the PARKIN protein level against 6-OHDA-induced decline. In this model, the protective mechanisms of Sal against 6-OHDA-induced cytotoxicity might involve in mitophagy, oxidative stress, or endoplasmic reticulum stress (Tao et al., 2016).

The UPS and ALP (the latter of which involves macroautophagy, microautophagy, and chaperone-mediated autophagy) are the two major degradation pathways maintaining intracellular proteostasis. The ALP is primarily responsible for the bulk degradation of redundant cellular materials under various stress conditions, including longer-lived macromolecules and damaged organelles (Klionsky, 2007; He and Klionsky, 2009). Meanwhile, the UPS is a highly selective and tightly regulated pathway for degradation of non-functional, potentially toxic soluble proteins, most of which are short-lived (Goldberg, 2003). Protein degradation by the UPS is ATP-dependent, and it involves two consecutive steps. The first step is ubiquitination in which a chain of activated ubiquitin monomers is linked to lysine residues in the substrate protein. This occurs via a cascade of enzymatic reactions mediated by E1-activating, E2-conjugating, and E3-ligating enzymes. In the second step, the ubiquitin chain is recognized by the proteasome and the substrates are degraded (Hershko and Ciechanover, 1998; Low, 2011; Komander and Rape, 2012). PARKIN, an E3-ligating enzyme, regulates mitochondrial quality control and protein ubiquitination. Mutations in the *PARKIN* gene are one of the most common causes of familial-recessive PD (Lucking et al., 2000; Mizuno et al., 2001). UCH-L1 is a highly abundant neuronal deubiquitinating enzyme that comprises 2% of the total protein in the brain. It regulates monoubiquitin stability and homeostasis in the nervous system, and its absence or dysfunction as a result of missense mutations is associated with neurodegeneration (Leroy et al., 1998; Saigoh et al., 1999). Cumulatively, the UPS represents a key mechanism for α-syn degradation (Cook and Petrucelli, 2009; Ebrahimi-Fakhari et al., 2011). Our study revealed that 6-OHDA impaired the UPS by reducing the proteins level of free ubiquitin, UCH-L1, and PARKIN, in addition to inhibiting proteasome activity. Sal recovered the UPS normal function. Using UPS and autophagy inhibitors, we showed that Sal decreased the pSer129-α-syn protein level induced by 6-OHDA in a UPS-dependent manner.

Substantial evidence suggests that missense point mutations (such as A53T, A30P, and E46K) or duplications in the α-syn-encoding *SNCA* gene lead to autosomal-dominant early-onset PD (Polymeropoulos et al., 1997; Kruger et al., 1998; Zarranz et al., 2004). In addition, overexpression of WT, A30P, or A53T α-syn in transgenic mice caused motor deficits and neurodegenerative alterations. Meanwhile, methods to reduce the accumulation of α-syn in the neurons of these mice inhibited the formation of neuronal inclusions, halting disease progression (Masliah et al., 2000; Freichel et al., 2007; Oaks et al., 2013; Diepenbroek et al., 2014). In the present study, 6-OHDA and increased levels of WT/A30P-α-syn impair proteasome activity and cell viability, these findings are consistent with previous studies (Alves et al., 2006; Jiang et al., 2007; Zhang N.Y. et al., 2008; Ebrahimi-Fakhari et al., 2011). Inhibiting proteasome function using MG132, Sal-mediated clearance of α-syn was lost and cell viability decreased significantly. Luciferase reporter gene assay showed that MG132 did not activate the CMV promoter in WT/A30P-α-syn plasmids vector. These data revealed that Sal promoted WT/A30P-α-syn clearance by enhancing 20S proteasome activity. In addition to pSer129-α-syn, which is targeted by the UPS (Machiya et al., 2010), WT-α-syn and mutant A30P/A53T-α-syn are degraded by both the UPS and ALP (Webb et al., 2003; Lan et al., 2012; Yang et al., 2013). After inhibiting autophagy, we showed that Sal protected cells and promoted the clearance of WT/A30P-α-syn mainly via the UPS.

## Conclusion

Our studies demonstrated for the first time that Sal decreased the pSer129-α-syn level and removed WT/A30P-α-syn by maintaining the UPS normal function in SH-SY5Y cells. This suggests that Sal may act as a potential neuroprotective agent that reverses the abnormal UPS. Sal should be tested in animal models mimicking the progression of PD to assess its potential as a candidate for a clinical trial.

## Author Contributions

TL, JC, and QY designed the study. TL, YF, RY, LH, and LW performed the major experiments. RL tested the 20S proteasome activity. TL, YF, JC, and QY analyzed the data and discussed the results. TL, YF, and JC wrote the manuscript. All authors approved the final version of the manuscript.

## Conflict of Interest Statement

The authors declare that the research was conducted in the absence of any commercial or financial relationships that could be construed as a potential conflict of interest.
