# Supplementary material for: Salidroside Promotes the Pathological α-Synuclein Clearance Through Ubiquitin-Proteasome System in SH-SY5Y Cells
Source: Front Pharmacol. 2018 Apr 19;9:377. doi: 10.3389/fphar.2018.00377 (PMC5917065; doi:10.3389/fphar.2018.00377)
Supplement: Supplementary file 1 [file Data_Sheet_1.doc]

**Salidroside promotes the pathological α-synuclein clearance through Ubiquitin-proteasome system in SH-SY5Y cells**

Tao Li*^1^, Yang Feng*^1^, Ruixin Yang^2^, Leitao Wu^1^, Ruru Li^1^, Lu Huang^2^, Qian Yang^2^ and Jianzong Chen^1^

**Authors’ Affiliations**

^1^Research Center of Traditional Chinese Medicine, Xijing Hospital, The Fourth Military Medical University, 169 West Changle Road, Xi'an, Shaanxi 710032, China

^2^Department of Neurosurgery, Tangdu Hospital, The Fourth Military Medical University, 569 Xinsi Road, Xi'an, Shaanxi 710038, China

*These authors contributed equally to this study

**Corresponding Author**

Qian Yang: Tel:+86029-84717824. E-mail address: qianyang@fmmu.edu.cn.

Jianzong Chen: Tel:+86029-84775955. E-mail address: jzchen57@fmmu.edu.cn.


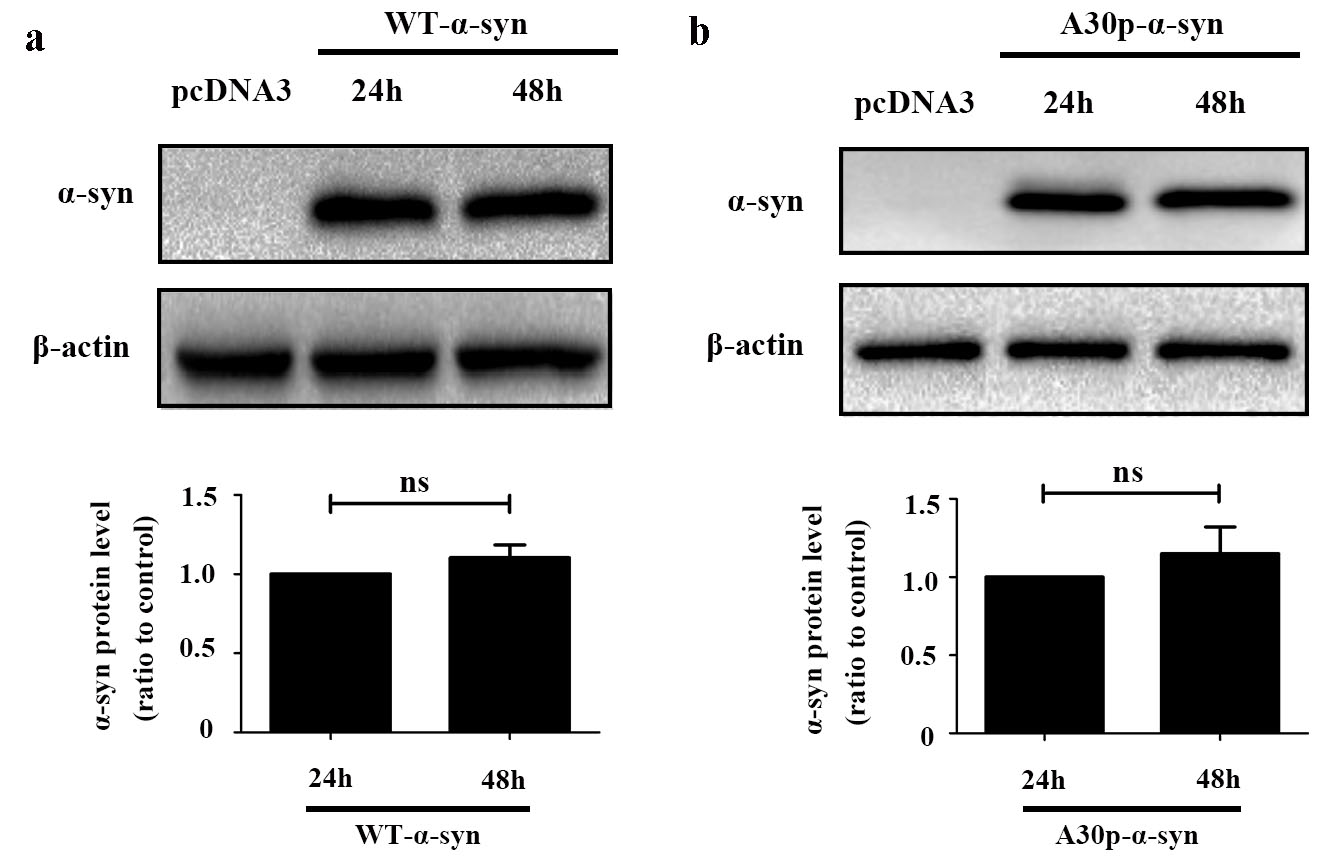


**Supplementary Figure 1. WT/A30P-α-syn protein were stably expressed in WT/A30P-α-syn plasmids transfected-cells between 24 h and 48 h.**

(a-b) SH-SY5Y cells were transfected with WT/A30P-α-syn plasmids for 24 h and 48 h, then the α-syn protein levels were investigated by Western bloting. Data are shown as the mean±SEM. Statistical significance was analyzed by one-way analysis of variance (ANOVA). A 2-tailed Student’s t test was performed for comparisons between two groups. Each experiment was repeated three times independently. ***** P<0.05; ****** P<0.01, ns: no significance.
